# Hypertriglyceridemic pancreatitis managed with heparin and insulin: a case report

**DOI:** 10.1186/s13256-023-03995-x

**Published:** 2023-06-20

**Authors:** Zablon Mesfin Anbessie, Yohannes Birhanu Gebremeskel

**Affiliations:** 1 Bethzatha General Hospital, P.O. Box 57060, Addis Ababa, Ethiopia; 2 College of Health Sciences, Addis Ababa University, Addis Ababa, Ethiopia

**Keywords:** Acute pancreatitis, Hypertriglyceridemia, Insulin, Heparin, Case report

## Abstract

**Background:**

Alcohol and gall stones are common causes of pancreatitis. Other causes of pancreatitis include hypertriglyceridemia, trauma, congenital anomalies, and medications. Hypertriglyceridemic pancreatitis is distinguished, as it is more severe and complicated. The management of hypertriglyceridemic pancreatitis, other than the basic care given to other pancreatitis patients, is to decrease the serum triglyceride level to less than 500 mg/dl as soon as possible. Plasmapheresis, hemofiltration, and other modalities have been proven effective therapies, but, are expensive and not easily accessible. Insulin and heparin which are cheaper alternatives for treatment, have been reported in case reports along with one randomized controlled trial. The number of patients in these reports was small, so, the therapy is not well established. For most African countries like ours, the only option for management is heparin and insulin. Despite this fact, there has not been any publication regarding this issue on our continent.

**Case report:**

We report the case of a 24 years old Ethiopian male who presented with severe central abdominal pain, easy fatiguability, and vomiting of one-day duration. He was tachycardic and tachypneic with diffuse abdominal tenderness, and had tendon xanthomas. His plasma was lactescent with a serum triglyceride level of 4775 mg/dl. His abdominal CT scan showed diffuse pancreatic swelling with a peripancreatic fluid collection, and his serum lipase was elevated. With a diagnosis of hypertriglyceridemic pancreatitis, he was managed with intravenous insulin infusion along with subcutaneous heparin. His random blood sugar was checked hourly with three episodes of hypoglycemia during therapy. His serum triglyceride level dropped to less than 500 mg/dl in three days, and he was discharged with no complications.

**Conclusion:**

Since our findings are consistent with a prior randomized controlled trial and compilation of case reports, it would strengthen the evidence for safety and efficacy of insulin and heparin therapy. This therapy, which is the only available therapy in most countries of our continent, would decrease most of the complications of hypertriglyceridemic pancreatitis that we face. We believe, our report would be a wake-up call for researchers and clinicians in our continent to change their practice and strengthen the evidence for the treatment.

## Introduction

Acute pancreatitis (AP) is inflammatory damage to the pancreas. It causes a set of clinical symptoms and signs, with serum amylase levels being elevated three times the upper limit of normal, and imaging that shows evidence of inflammation in the pancreas. The global incidence of acute pancreatitis has been estimated to be 33.74 per 100,000 population, while its mortality has been estimated to be 1.16 per 100,000 population [1–7].

The most common causes of acute pancreatitis are gall stones and alcohol. While other less common causes include hypertriglyceridemia (HTG), trauma, medications, and congenital anomalies [3]. Among these causes, HTG tends to cause more severe and complicated acute pancreatitis, so, it needs to be considered early in the management of any patient with AP. Hyertriglyceridemic pancreatitis (HTGP) accounts for 1–10% of the cases of AP [1, 3, 4, 6, 8].

Hypertriglyceridemia can occur either as a primary disorder (type I, IV, and V hyperlipoproteinemia), or following alcoholism, diabetes, pregnancy, obesity, and the use of some drugs. Seventy-five percent of hypertriglyceridemic patients, either have uncontrolled diabetes or are alcoholics [6]. When serum triglyceride (TG) level is elevated, the pancreatic and endogenous lipases hydrolyze TG molecules to free fatty acids and glycerol. The free fatty acids that are released into the blood will be bound by albumin until all the albumin is saturated. When saturation occurs, the free fatty acids in serum cause cytotoxic injury to the endothelium of the pancreatic circulation causing stasis of blood, pancreatic ischemia, and inflammation [3, 6].

A serum TG level above 1000 mg/dl increases the risk of AP to 16–20%. The risk increases even further by 4%, for every 100 mg/dl rise in serum TG level above 1000 mg/dl. In patients with AP, the presence of a lactescent serum is strongly correlated with HTGP [3, 4, 6, 8]. In patients less than 50 years of age HTGP is more common in males. After the recovery of HTGP serum TG levels appear to correlate with recurrence [3, 6].

The management of HTGP includes; keeping patients fasted, analgesics, and fluid resuscitation along with treatments targeted to acutely decrease the serum TG level below 500 mg/dl. To attain these goals, plasmapheresis, insulin infusion, heparin, fibrate therapy, and apolipoprotein C-II derivatives have been used [5, 6]. Plasmapheresis is expensive, inaccessible, and lacks strong evidence for improving morbidity and mortality, despite its efficacy in lowering serum TG levels to target within 3 h. Fibrate therapy is reserved for the chronic management of HTG, and apolipoprotein C-II derivatives are difficult to access and are less used [3–5].

The use of insulin alone or in combination with heparin, even though cheap and easily accessible, has not been definitively established in the management of HTGP. Heparin and insulin enhance the activity of lipoprotein lipase and increase its release from the endothelium, aiding in the lowering of serum TG levels. In all cases, insulin is used as an infusion while heparin is administered subcutaneous (SC) or intravenous (IV) at varying doses. Both unfractionated and low molecular weight heparin has been used in the literature, and both seem to have equal efficacy [3, 5, 6, 8].

One randomized controlled trial and some case series have shown the safety and efficacy of heparin and insulin in the management of HTGP. To the author's knowledge 56 cases of HTGP managed with insulin and heparin have been reported so far, 22 from case compilations and 34 from a randomized controlled trial reported from China. There has been no publication regarding the use of insulin and heparin from the African continent, where all the other management modalities are either expensive or not available. This report specifically hopes to show that a cheap, effective, and safe therapy for HTGP is available, and can be used in the African continent.

### Case report

A 24 years old nonalcoholic male Ethiopian, with no prior comorbidities, presented to the emergency department (ED) complaining of; severe central abdominal pain, easy fatiguability, and vomiting of one-day duration. He had been complaining of increased thirst, water intake, and urination for the past one week before his presentation. He was told to have a high cholesterol level and was on dietary management for the past three years.

On examination, he had a pulse rate of 120 beats per minute, a respiratory rate of 28 breaths per minute, a blood pressure of 160/100 mmHg, a temperature of 36 °C, oxygen saturation of 88% on room air, and a Body mass index of 26.4 kg/m^2^. He had right posterior basal third crepitation and decreased air entry, suggestive of atelectasis with reactive effusion. The abdomen was diffusely tender with increased tenderness over the epigastric area. There were also nodular firm lesions over the extensor aspect of both elbows, suggestive of tendon xanthomas.

His investigations showed that he had a leukocytosis of 21,700 cells/mm^3^, hemoglobin of 14 g/dl, and a platelet count of 214, 000 cells/mm^3^. He had a serum triglyceride level of 4775 mg/dl, a serum cholesterol level of 1080 mg/dl, and a low-density lipoprotein level of 395 mg/dl. His arterial blood gas was significant for metabolic acidosis with a serum PH of 7.34, bicarbonate of 18.9 mmol/L, partial pressure of carbon dioxide of 35 mmHg, and serum lactate of 3.67 mg/dl. His serum amylase and lipase levels were elevated at 1136 and 1687 U/L respectively. His admission random blood sugar was 313 mg/dl with a urine ketone level of + 2 and a HgbA1c of 8. His renal function test, transaminase levels, and electrolytes were in the normal range on admission (Table 1).

**Table 1:** Abnormal laboratory parameters

Laboratory parameter	Result on admission	30 h later	78 h later
White cell count (Cells/mm^3^)	21,700	–	–
Platelet (Cells/mm^3^)	214,000	–	–
Triglyceride (mg/dl)	4775	720	350
Total cholesterol (mg/dl)	1080	353	296
High density lipoprotein (mg/dl)	35	38	42
Low density lipoprotein (mg/dl)	395	171	184
PH	7.34	–	–
HCO3(mmol/L)	18.9	–	–
PCO2(mmHg)	35	–	–
Serum lactate (mg/dl)	3.67	–	–
Lipase (U/L)	1687	–	–
Amylase (U/L)	1136	–	–
Random blood sugar (mg/dl)	313		
Urine ketone	+ 2		

*PH* Potential of hydrogen,* HCO3* serum bicarbonate,* PCO2* partial pressure of carbon dioxide

**Fig. 1:**
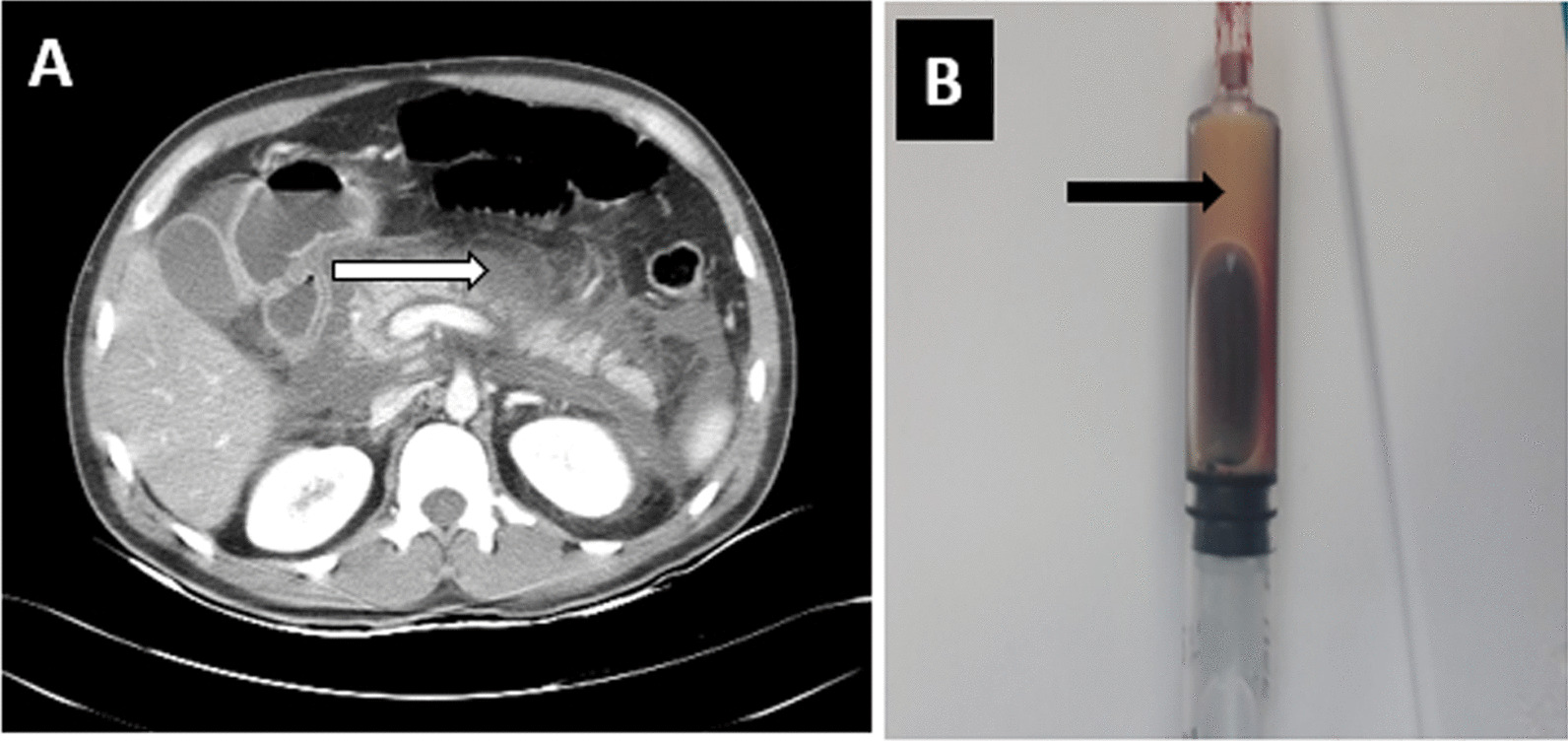
**A** Diffusely swollen pancreas with peri-pancreatic fat stranding and Peri-pancreatic fluid (white arrow) and **B** lactescent serum from a blood sample (black arrow)

His admission abdominal ultrasound showed a diffusely hypoechoic pancreas with peripancreatic fat stranding. An abdominal CT scan with contrast done two days later showed a diffusely swollen pancreas with poor contrast uptake and peri-pancreatic fat stranding with a peri-pancreatic fluid collection. As there were financial and investigation access limitations, we failed to perform genetic tests for hyperlipidemia (Fig. 1).

He was admitted to the ICU with a diagnosis of severe HTGP and diabetic ketoacidosis, and an intravenous loading dose of regular insulin at 0.1 IU/Kg was given, followed by an hourly IV infusion of 0.1 IU/Kg every hour. He was also put on a standing dose of morphine, six bags of IV normal saline, and heparin 5000 IU SC twice a day. He had an hourly measurement of his blood sugar, with two hourly measurements of his urine ketone. He had required infusions of 500 ml, 5% dextrose in water, mixed with 500 ml of normal saline in one bag, along with intravenous potassium chloride, when his random blood sugar dropped below 250 mg/dl, and his serum potassium dropped below 5.3 meq/L. He had three episodes of hypoglycemia at 62,69 and 54 mg/dl during his management, which required 1.25 ml/kg Iv push of 40% dextrose. He was taken out of diabetic ketoacidosis in 30 h, and the serum TG level had dropped below target, within three days of his treatment. He was discharged one week after admission, with Omega 3 fatty acids, gemfibrozil, and Neutral protamine Hagedorn insulin.

He had two follow-ups in three months during which he had; good functional status, no abdominal complaints, serum TG of 250 mg/dl, and no local pancreatic complications on a repeat abdominal CT scan.

## Discussion

We were able to show that, insulin infusion and a prophylactic dose of heparin, when given subcutaneously along with the standard management of AP, were able to decrease the serum level of TG in a patient with HTGP. This was achieved within three days of therapy, with no significant harm to our patient.

All the case series and case reports on HTGP had described patients in their young and middle age (28–55 years), who had presented to a hospital with epigastric pain, within three days of their illness. More than 85% of the patients in these reports were males [5, 6, 9, 10]. In an open-label randomized controlled trial done in China; the mean age at presentation was 41 ± 8 years (18–85), with 23% of the patients being females, and 18% being diabetics. These background characteristics in the literature are similar to our patient. Even though we were unable to perform further genetic diagnostic tests, the patient had tendon xanthomas that may be related to underlying genetic HTG.

Most of the patients described in the literature had attained a TG level target of less than 500 mg/dl, at or beyond day three of their treatment. Most of the publications had used insulin infusion along with a prophylactic dose of subcutaneous heparin. We were able to achieve the same results in our patient with insulin infusion and a prophylactic dose of heparin. His serum TG level fell from 4775 mg/dl to 350 mg/dl by day three [4–6, 8, 9] (Table 2).

**Table 2:** Comparison of our patient’s Triglyceride level and Insulin heparin protocol to prior studies

Publication	Patient No	Serum triglyceride (mg/dl)			Insulin	Heparin
		Day 1	Day 2	Day 3		
Jain *et al.* [5]	2	1808 3743	867 1804	570 1015	Infusion	UFH SC
Alagözlü *et al.* [6]	1	1,707	713	589	Infusion	UFH SC
Gennis *et al.* [8]	1	10,560	1479	712	Sliding scale	Not administered
Kuchay *et al.* [7]	4	1800–5900	NA	500–1600	Infusion	HD UFH IV
He *et al.* [4]	34	1966 ± 806	449 ± 189	NA	Infusion	UFH SC
Our patient	1	4775	720	350	Infusion	UFH SC

*UFH SC* Unfractionated heparin subcutaneously, *NA* Not available, *HD UFH IV* High dose unfractionated heparin intravenously

A cohort study of patients with acute pancreatitis from New Zealand had shown that the use of insulin during or after the treatment of acute pancreatitis, had increased the risk of developing recurrent acute pancreatitis and chronic pancreatitis by 70% over a mean follow-up period of seven years. We were unable to perform this comparison as the patient was lost to follow-up after three months [11].

## Conclusions

Based on our case report and analysis of the literature reported so far, we propose that the use of insulin infusion along with a prophylactic dose of heparin can be used in our country and the African continent, where the availability and cost of the other therapies for HTGP make their use impossible.

## Data Availability

All have been included in the manuscript.

## Abbreviations

AP: Acute pancreatitis
ED: Emergency department
HTG: Hypertriglyceridemia
HTGP: Hypertriglyceridemic pancreatitis
IV: Intravenous
SC: Subcutaneous
TG: Triglyceride
